# A plasmid containing CpG ODN as vaccine adjuvant against grass carp reovirus in grass carp *Ctenopharyngodon idella*

**DOI:** 10.18632/oncotarget.21245

**Published:** 2017-09-23

**Authors:** Hang Su, Zhiwei Liao, Gailing Yuan, Jianguo Su

**Affiliations:** ^1^ College of Fisheries, Huazhong Agricultural University, Wuhan, China; ^2^ Hubei Engineering Technology Research Center for Aquatic Animal Disease Control and Prevention, Wuhan, China; ^3^ Laboratory for Marine Biology and Biotechnology, Qingdao National Laboratory for Marine Science and Technology, Qingdao, China

**Keywords:** grass carp (*Ctenopharyngodon idella*), plasmid containing CpG ODNs, grass carp reovirus, adjuvant, immune protection

## Abstract

CpG oligodeoxynucleotides (ODNs) are proved to have strong immune stimulatory activity. Plasmids containing CpG ODNs could be conveniently and low-costly used as vaccine adjuvant. However, they are different among various plasmids, motif repeats, species, etc. In the present study, plasmid pcDNA3.1 (+) containing five repetitions of CpG ODN 1670A named pcDNA3.1-1670A*5 with strong immunostimulation was screened out from twelve recombinant plasmids and three empty vectors by cell proliferation activity, interferon promoter activities and immune related gene expressions in CIK cells. It works through TLR9-mediated signaling pathway, triggering the immune related genes expression. Furthermore, the potentiality of pcDNA3.1-1670A*5 as adjuvant was tested *in vivo*. pcDNA3.1-1670A*5 was co-inoculated with inactivated GCRV vaccine on grass carp fingerlings. Immunoglobulins (IgM, IgD, IgZ), TLR9, IFNγ2, IFN1, TNF-α, Mx2 and VP4 were examined. Ultimately, pcDNA3.1-1670A*5 significantly enhanced the expressions of IgM in serum, head kidney and spleen, recognition receptor TLR9 as well as antiviral effector molecule Mx2, and inhibited GCRV proliferation in head kidney and spleen tissues. The present study explored the application and mechanism of plasmid containing CpG ODN as high-efficient adjuvant to promote efficiency of vaccine and control disease in grass carp, which will contribute to the development of new type CpG ODN adjuvant in aquaculture industry.

## INTRODUCTION

Grass carp reovirus (GCRV) is recognized as the most virulent agent among all of the identified isolates in the genus *Aquareovirus* of the family Reoviridae [1]. It is a fatal pathogen to aquatic animals which can provoke severe hemorrhagic disease in fingerling and yearling populations of grass carp causing a mortality rate of up to 85% during an outbreak [2]. Grass carp (*Ctenopharyngodon idella*), a fish species of the largest production in the world, is one of the most important freshwater aquatic animals in China and a significant economical species extensively farmed in many Asian countries [3]. To control the spread of grass carp hemorrhagic disease, several vaccines have been developed over the years. Vaccination is an effective method in decreasing the incidence of infectious diseases caused by pathogens [4]. Grass carp hemorrhagic disease vaccine mainly focuses on inactivated vaccine and attenuated vaccine but their immune protective activity remains to be further studied. Vaccine is generally co-applied with an adjuvant to enhance immune responses [5, 6]. Adjuvant is a kind of compound which can strengthen the ability of the co-inoculated antigens to elicit early, long-lasting and high level immune responses [7]. Traditional adjuvant like freund’s adjuvant can make vaccines long-lasting and new type immune adjuvant which have immunogenicity like cytokine adjuvant can enhance immune responses.

CpG oligodeoxynucleotide (ODN), a new type immune adjuvant, contains dinucleotides with unmethylated CpG motifs and have been studied for the protective immunity against viral, bacterial, and parasitic infections in a large number of animal models [8–10]. It has shown strong immune stimulatory activity due to the common appearance of unmethylated CpG motifs in genomes of microbial pathogens while much less frequency in vertebrate genomes [11–14]. Because of the effect on stimulation of immunity, many completed clinical trials have suggested a promising application of CpG ODN as adjuvant [15]. Owing to their immune stimulatory activity, artificial synthetic CpG ODNs have been used as immune protective agents and adjuvants to mediate protective immune responses to various cancers, allergies and infectious diseases, especially those caused by viruses, such as respiratory syncytial virus [16], swine-origin influenza virus [17], hepatitis C virus [18] and human immunodeficiency virus [19]. To date, the majority of studies on CpG ODN were carried out on mammals in a variety of species protecting them from bacterial, viral and protozoan pathogens [13, 20]. Interestingly, plasmid containing CpG ODN is also proved to be useful as adjuvant for DNA vaccines. Co-administering CpG-enriched plasmids with a DNA vaccine encoding the envelope glycoprotein of HIV to BALB/c mice significantly increased HIV-specific cell mediated and humoral immunity [21, 22]. The adjuvant property of plasmid DNA was dependent on its content of CpG motifs which interacted with toll-like receptor 9 (TLR9) and directly induce the production of IL-12 and IFN (interferon)-α in dendritic cells [13, 23, 24]. TLR9, a pattern recognition receptor (PRR), has been proved to be the receptor of CpG ODN [25, 26]. CpG ODN could be recognized by TLR9 to promote the maturation of antigen presenting cells (APCs) and secretion of cytokines including IFN, interleukin (IL), tumor necrosis factor (TNF) and so on [27]. Subsequently, adaptive immune response is triggered. As one of the most important antibodies against pathogens in teleost, IgM is the primary immunoglobulin mediating humoral adaptive immunity in fish [28]. Therefore, IgM indicates the humoral immune responses in fish and identifies specific antigens for vaccines.

Our previous study showed that CpG ODN 1670A could act as an efficient immunostimulation in grass carp against GCRV [26]. However, the high cost of artificial synthesis of CpG ODN confines the application in grass carp cultivation. In this study, we constructed twelve plasmids containing CpG ODN sequences and screened out a suitable plasmid as adjuvant. The present study will facilitate the development of immune adjuvant for other species and lied the foundation for the application of CpG ODN in vaccine adjuvant.

## RESULTS

### pcDNA3.1-1670A*5 has the strongest proliferation enhancing activity among fifteen plasmids in CIK cells

**Figure 1:**
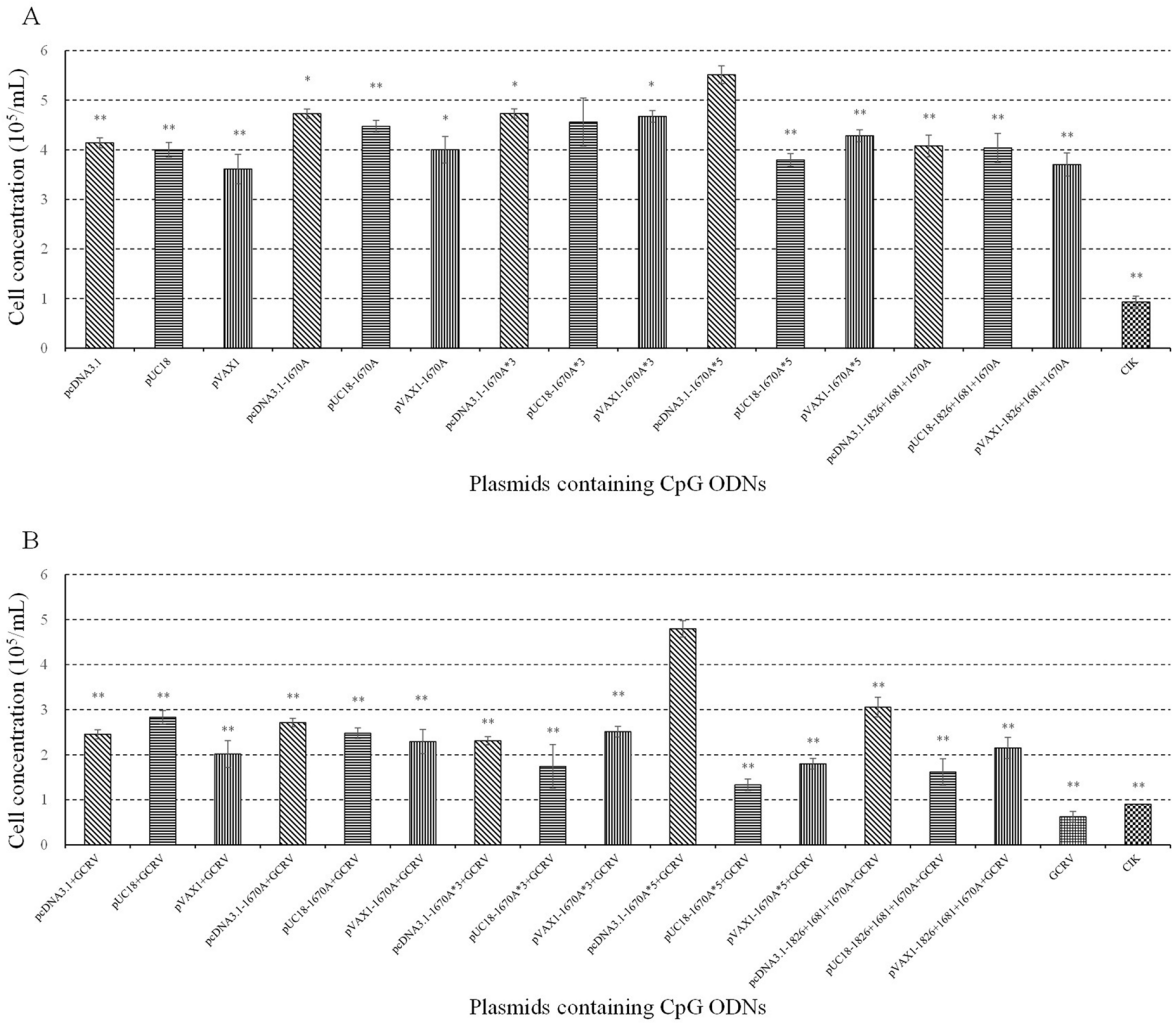
Effect of plasmids containing CpG ODNs on CIK cells **(A)** CIK cells were treated with PBS (control) or different plasmids and determined the proliferation activity by cell counting at 48 h post-stimulation. **(B)** CIK cells vaccinated with plasmids for 48 h were challenged with GCRV and determined the proliferation activity by cell counting at 24 h post-infection. Data are presented as means ± SE (n=3). The *p* value was calculated by student's t-test between pcDNA3.1-1670A*5 stimulation group and other groups (*, *p* ≤ 0.05, **, *p* ≤ 0.01). Error bars represent standard errors.

To characterize the effect of plasmids containing CpG ODNs on immune response enhancing activity and screen out the optimum one, proliferation of CIK cells was detected by cell counting. As shown in Figure 1A, most plasmids induced CIK cells proliferation, quantity of cells being displayed by cell concentration. pcDNA3.1-1670A*5 had the strongest proliferation enhancing activity.

### pcDNA3.1-1670A*5 has the best potential of being used as adjuvant against GCRV

To verify the potentiality to be used as vaccine adjuvant for application in antiviral immunity against GCRV in grass carp, antiviral activity was measured after vaccination and GCRV infection by cell counting, qRT-PCR and crystal violet staining. As shown in Figure 1B, after GCRV infection, cell concentration of each experimental groups decreased, while CIK cells treated with pcDNA3.1-1670A*5 still kept in high level indicating the strongest antiviral activity protecting CIK cells from infection among the twelve recombinant plasmids.

**Figure 2:**
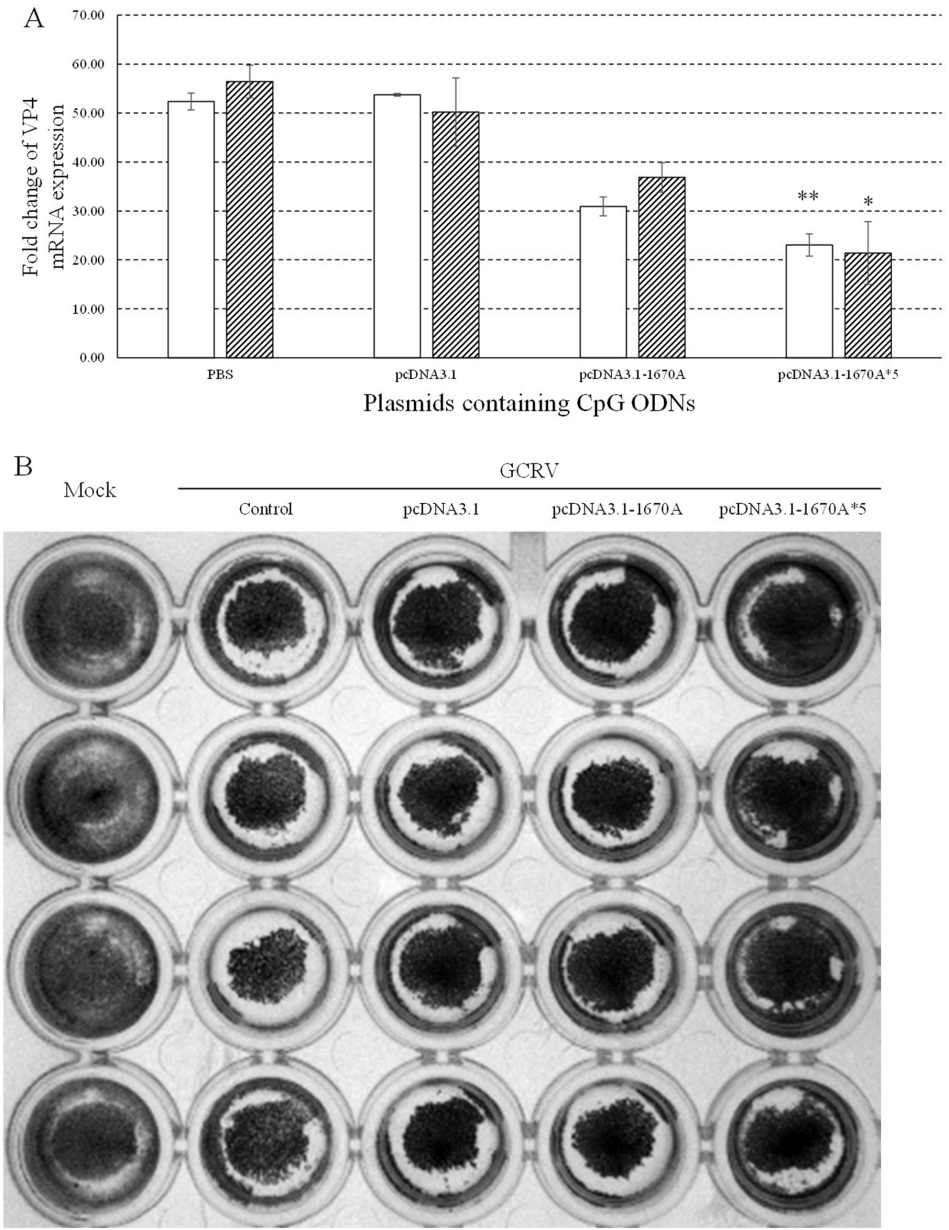
Antiviral activities of plasmids containing CpG ODNs in CIK cells **(A)** CIK cells were stimulated with PBS (control) or plasmids containing CpG ODNs and subsequently challenged with GCRV at 48 h post plasmids administration. The mRNA expression levels of VP4 were examined at 0, 24 and 48 h post-challenge. The EF1α gene was used as an internal control to normalize the cDNA template. mRNA levels of the target gene were normalized to those in CIK cells at 0 h. The *p* value was calculated by student's t-test between experimental and control groups (*, *p* ≤ 0.05, **, *p* ≤ 0.01). Error bars indicate SE (n = 3). Blank columns for 24 h and shadowy for 48 h. **(B)** Crystal violet staining of CIK cells after plasmids containing CpG ODNs stimulation and GCRV infection at 48 h post plasmids stimulation. Control group was treated with PBS and subsequently GCRV while mock group without any treatment.

VP4 transcript of GCRV was examined by qRT-PCR at 0, 24 and 48 h and normalized to EF1α expression levels to exclude the influence of cell concentration. Low VP4 expression indicates high antiviral activity of plasmids. As shown in Figure 2A, the expression of VP4 was repressed in different degrees. The strongest inhibition was seen in CIK cells stimulated with pcDNA3.1-1670A*5 and subsequent GCRV infection which showed extremely significant difference at 24 h and significant difference at 48 h. As shown in Figure 2B, the result of crystal violet staining showed that CIK cells in 96-well plate treated with pcDNA3.1-1670A*5 and subsequent GCRV infection remained the most quantity of cells. These observations showed that pcDNA3.1-1670A*5 could highly protect CIK cells from GCRV infection indicating its strongest antiviral activity.

### pcDNA3.1-1670A*5 promotes immune responses in CIK cells

**Figure 3:**
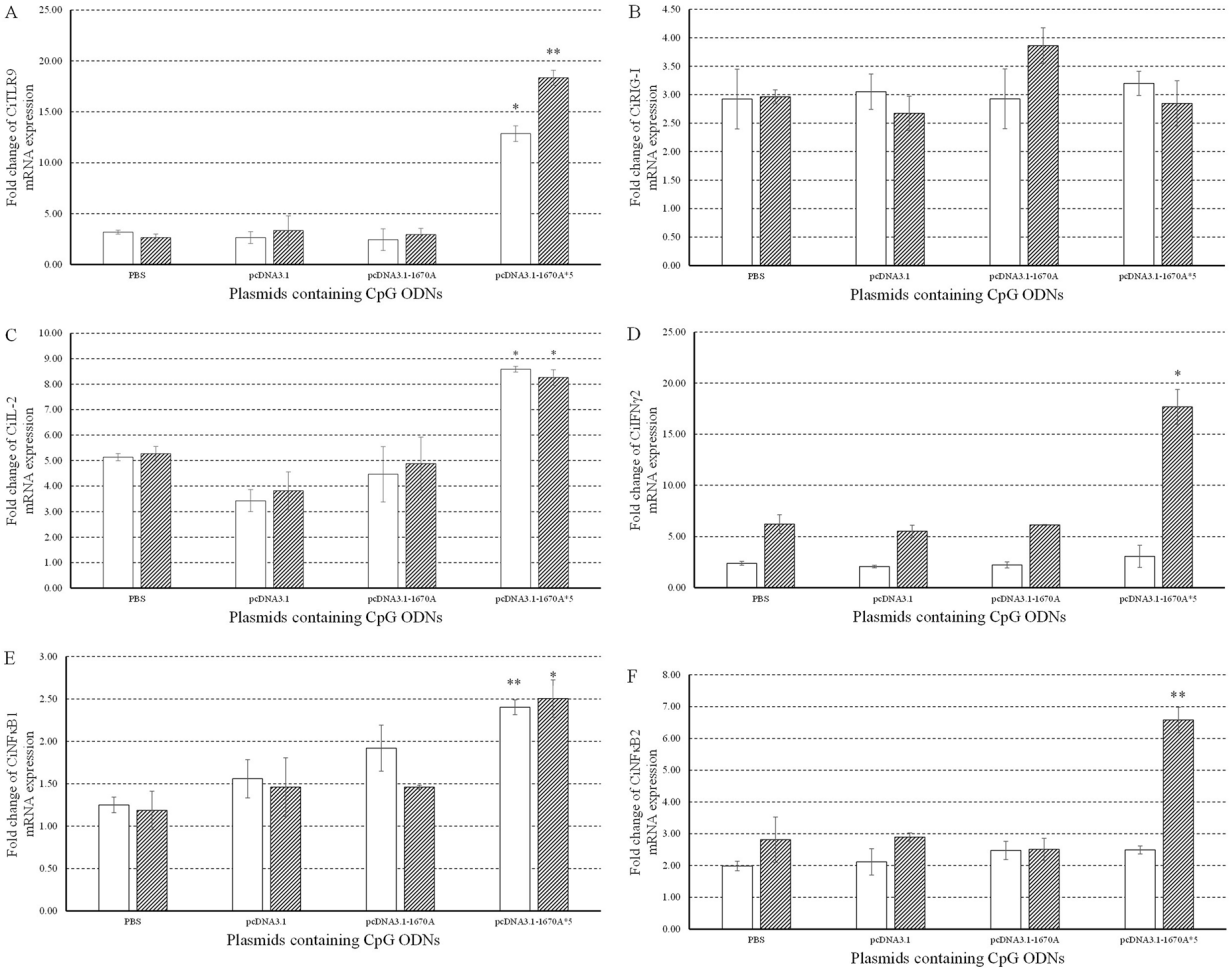
Immune responses to plasmids containing CpG ODNs in CIK cells The mRNA expressions of CiTLR9 **(A)**, CiRIG-I **(B)**, CiIL-2 **(C)**, CiIFNγ2 **(D)**, CiNFκB1 **(E)** and CiNFκB2 **(F)** were measured at 0, 24 and 48 h post-infection. CIK cells were stimulated with PBS (control) or plasmids containing CpG ODNs and infected with GCRV. The EF1α gene was used as an internal control to normalize the cDNA template. mRNA levels of the target gene were normalized to those in CIK cells at 0 h. The *p* value was calculated by student's t-test between experimental and control groups at each time point (*, *p* ≤ 0.05, **, *p* ≤ 0.01). Error bars indicate SE (n=3).

**Figure 4:**
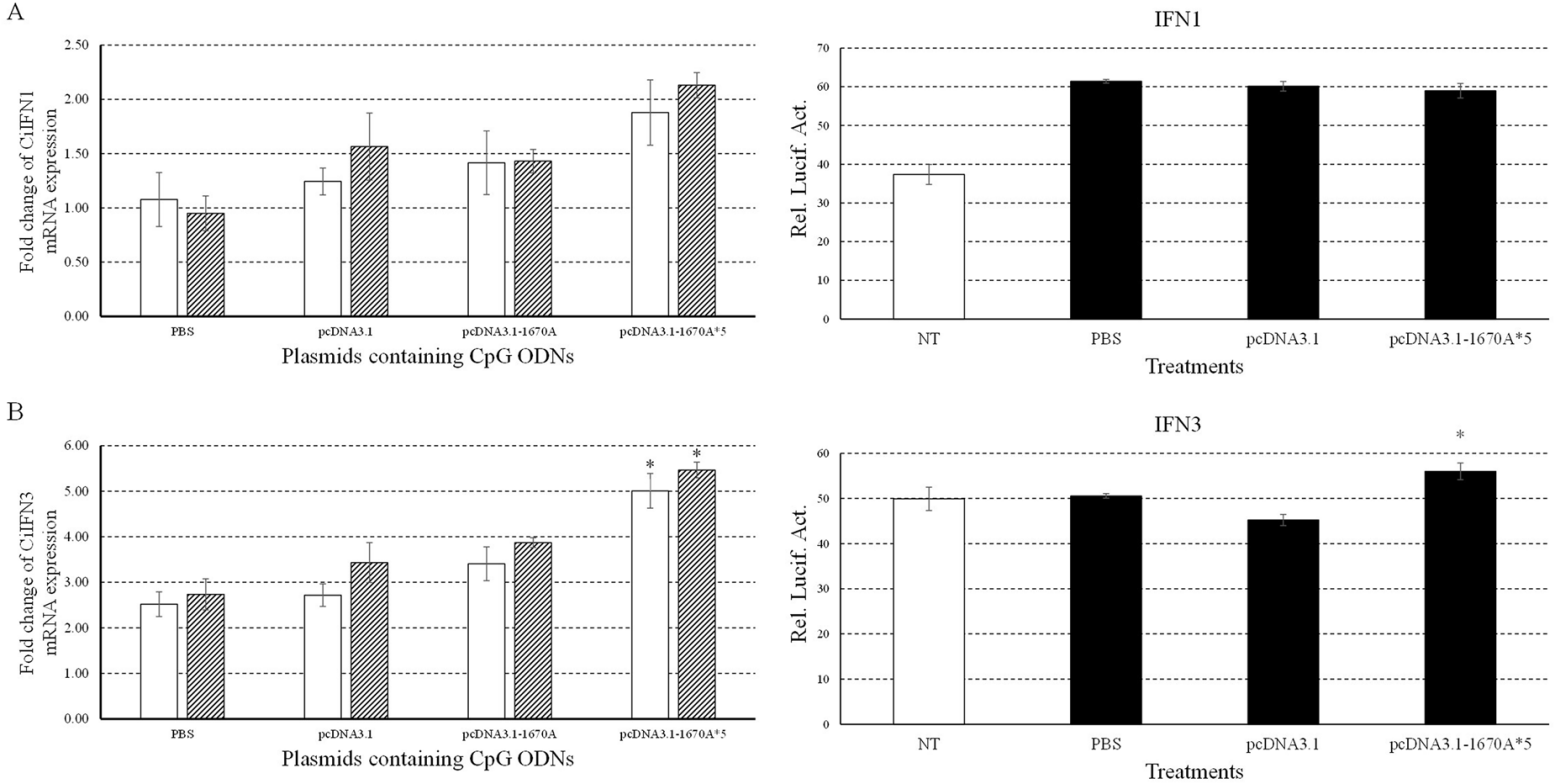
Immune responses of CiIFN1/3 to plasmids containing CpG ODNs Left, the mRNA expressions of CiIFN1 **(A)** and CiIFN3 **(B)** were measured at 24 and 48 h post-infection. CIK cells were stimulated with PBS (control) or plasmids containing CpG ODNs and infected with GCRV. Other captions were the same as Figure 3. Right, CIK cells were co-transfected with 800 ng of pRL-TK and IFN1pro-luc (A) or IFN3pro-luc (B) in 24-well plates. At 16 h post-transfection, the cells were stimulated with PBS (control) or plasmids and infected with GCRV for 16 h or uninfected. Dual luciferase reporter assays were conducted at 12 h after GCRV infection. Treatment durations in this assay was far shorter than those in qRT-PCR which caused different results. Error bars indicate standard deviation (n = 4). Asterisks indicate significant difference from control (*, *P* ≤ 0.05).

To investigate the immune responses to pcDNA3.1-1670A*5, the mRNA expression levels of CiTLR9 (Figure 3A), CiRIG-I (Figure 3B), CiIL-2 (Figure 3C), CiIL-12, CiIFNγ2 (Figure 3D), CiNFκB1 (Figure 3E), CiNFκB2 (Figure 3F), CiIFN1 (Figure 4A Left) and CiIFN3 (Figure 4B Left) were measured by qRT-PCR at 0, 24 and 48 h in CIK cells stimulated with PBS (control), pcDNA3.1 (+), pcDNA3.1-1670A or pcDNA3.1-1670A*5 for 48 h and subsequently challenged with GCRV. TLR9 is the specific receptor for CpG ODN [25]. CiTLR9, CiIL-2, CiIFNγ2, CiNFκB1, CiNFκB2 and CiIFN3 mRNA expression significantly increased upon pcDNA3.1-1670A*5 stimulation and GCRV infection in CIK cells at 24 and 48 h comparing to GCRV control, but did not after empty vector or pcDNA3.1-1670A treatment. Besides, CiRIG-I mRNA expression did not present any variation tendency and CiIFN1 was slightly induced by pcDNA3.1-1670A*5. The result of CiIL-12 mRNA expression was not shown because its inhibited expression cannot be detected by qRT-PCR.

Moreover, we also examined promoter activities of grass carp IFN1 (Figure 4A Right) and IFN3 (Figure 4B Right) by dual luciferase reporter system in CIK cells upon pcDNA3.1-1670A*5 vaccination and GCRV infection. Promoter activity of CiIFN3 was significantly enhanced by pcDNA3.1-1670A*5 stimulation and subsequent GCRV infection, while that of CiIFN1 did not respond differently comparing to other two experimental groups. The treatment duration of plasmid vaccination and GCRV infection in dual luciferase reporter experiment were different from that in sample of qRT-PCR.

### IgM expression is up-regulated by pcCpG *in vivo*

**Figure 5:**
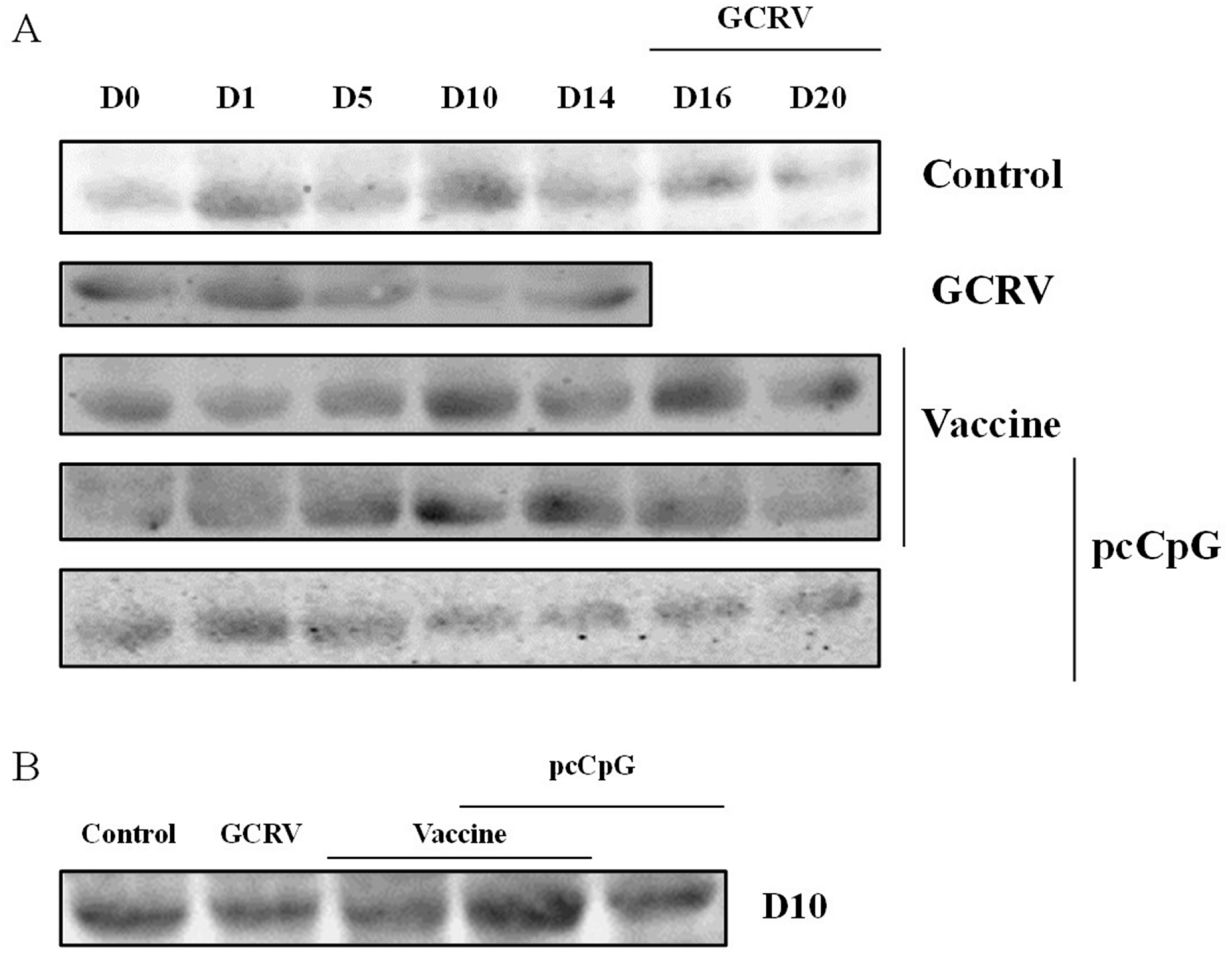
Serum IgM expression detection by western blot **(A)** Grass carp serum was obtained from grass carp vaccinated with NS, vaccine, vaccine-pcCpG or pcCpG and subsequently infected with GCRV at D0, D1, D5, D10, D14, D16 and D20. Grass carp serum of GCRV group was obtained at D16, D20, D25 and D29. **(B)** Expression difference among 5 groups of grass carp at D10.

To investigate the effect of pcCpG on CiIgM expression *in vivo*, serum of five random grass carp were sampled at each point-in-time to be detected by western blotting (Figure 5). In the same volume of serum, CiIgM expression of each group presented different tendency, while that of group vaccine-pcCpG gradually increased and reached the peak at D10 and kept in a high level (Figure 5A). At D10, CiIgM expression of group vaccine-pcCpG was obviously higher than other groups (Figure 5B) indicating that pcCpG effectively enhanced CiIgM expression assisting vaccine.

**Figure 6:**
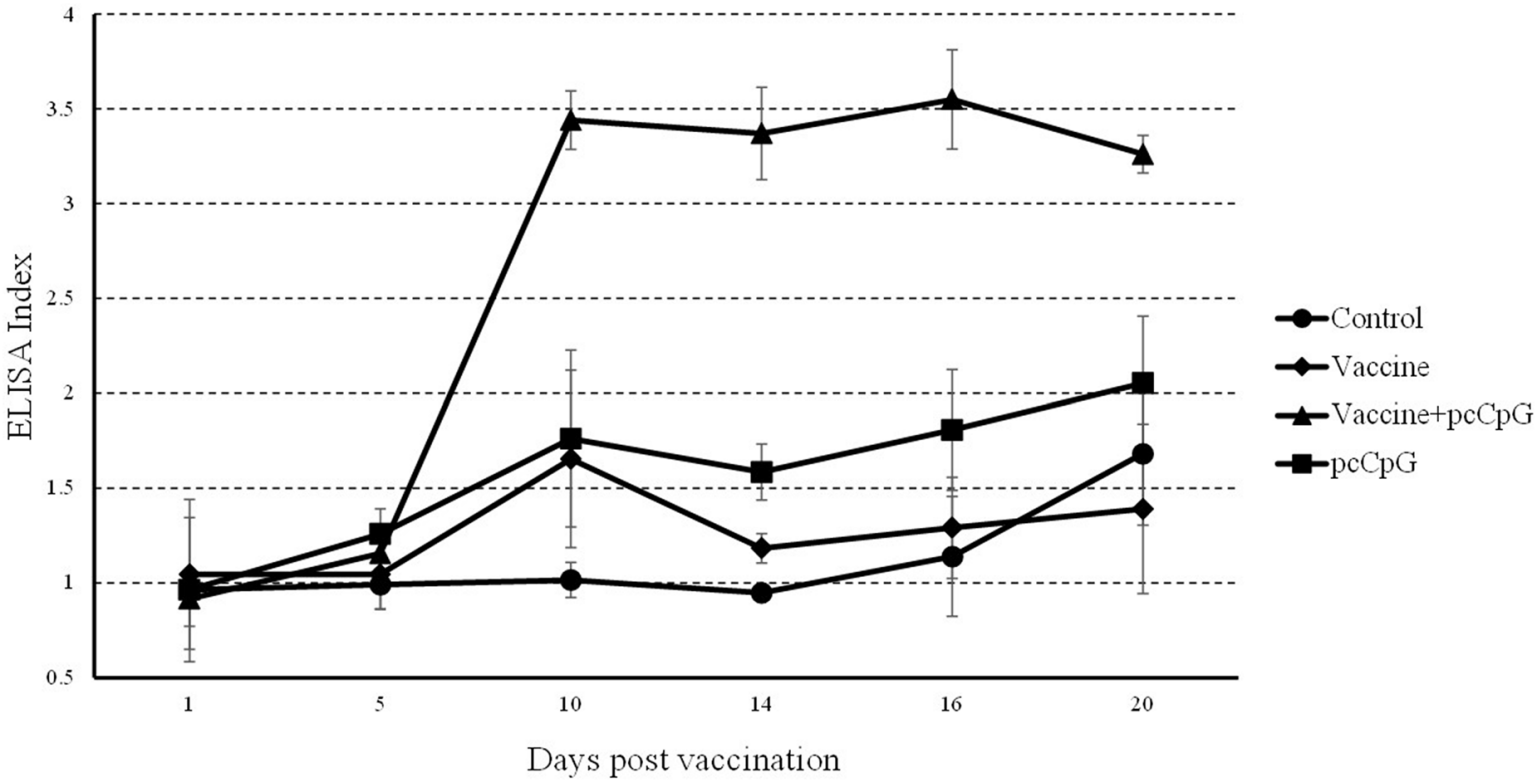
Serum IgM detection by ELISA Sera were collected at different days post immunization or GCRV infection from the fish vaccinated with PBS (control), vaccine, vaccine-pcCpG or pcCpG at D0, D1, D5, D10, D14, D16 and D20. The data of GCRV group were integrated to control group being showed as D16 and D20 in control group. ELISA index was normalized to D0. Data are presented as means ± SD (n = 5).

In the result of ELISA, the index of group vaccine-pcCpG drastically rose to about 3.5 at D10 and kept in a high level while those of other groups did not change that strongly (Figure 6).

**Figure 7:**
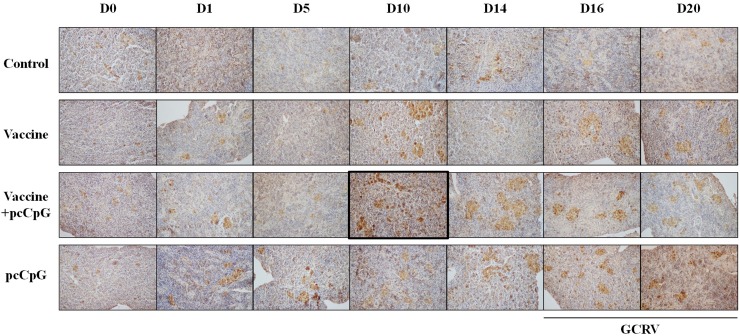
Immunohistochemistry analysis of IgM in spleen tissue of grass carp at D0, D1, D5, D10, D14, D16 and D20 post vaccination or subsequent infection The data of GCRV group were integrated to control group being showed as D16 and D20 in control group. Original magnification ×400.

For the IHC result, spleen tissues of each group were sampled at D0, D1, D5, D10, D14, D16 and D20 (Figure 7). The positive rate of group vaccine-pcCpG at D10 (marked in figure) was obviously higher than other groups at other point-in-times (Figure 7) indicating the highest CiIgM expression level.

### pcCpG activates numerous immune pathways *in vivo*

**Figure 8:**
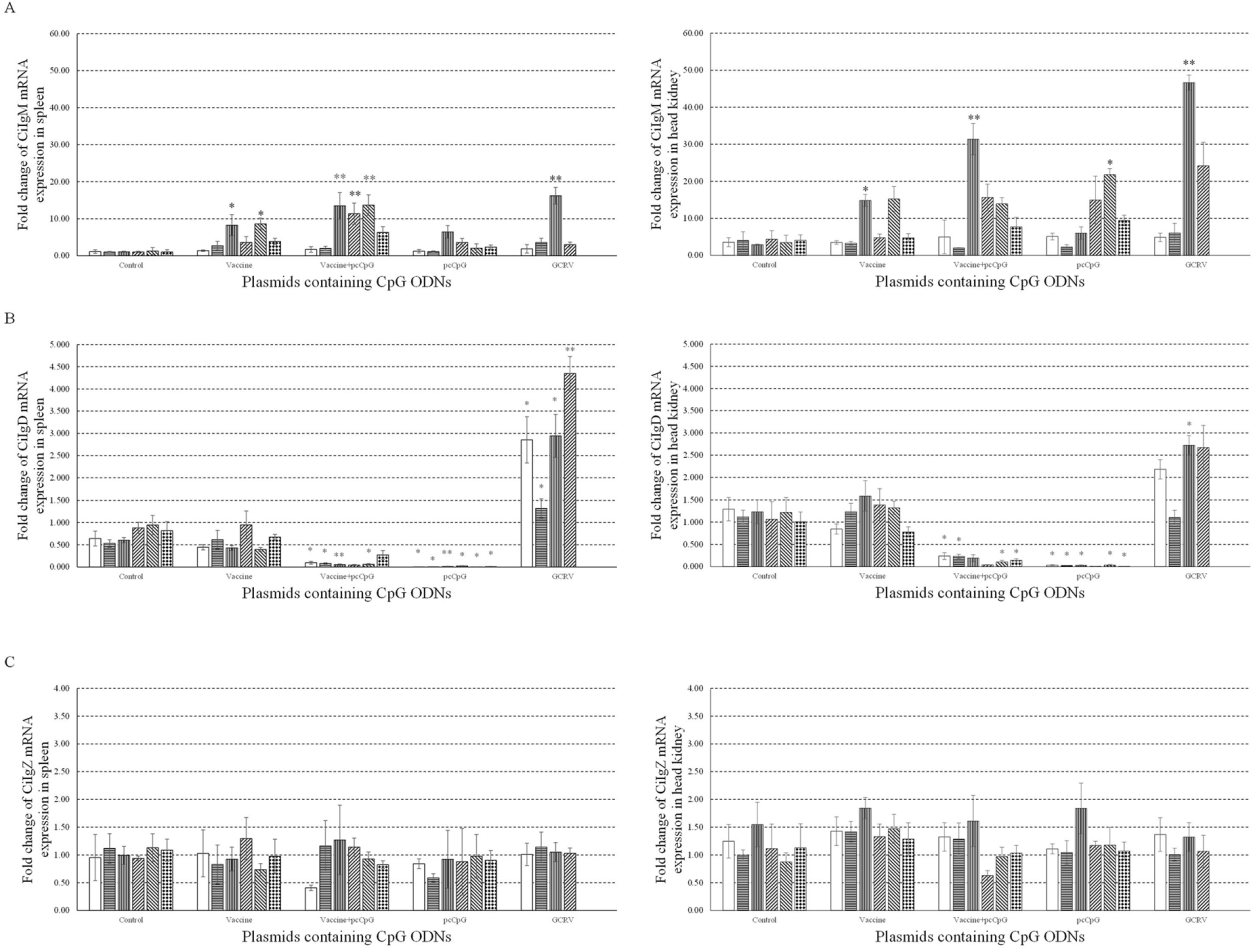
mRNA expressions of three immunoglobulins The mRNA expressions of CiIgM **(A)**, CiIgD **(B)** and CiIgZ **(C)** were measured at D0, D1, D5, D10, D14, D16 and D20 post immunization or subsequent infection in spleen (Left) or head kidney (Right). Grass carp were vaccinated with normal saline (NS), vaccine, vaccine-pcCpG and pcCpG respectively, and spleen and head kidney tissues were obtained from 5 random grass carp from each group at each time point. Different columns represent different time points. The 18S rRNA gene was used as an internal control to normalize the cDNA template. mRNA levels of the target gene were normalized to those in spleen tissue at 0 h. Asterisks (*) mark the significant difference (*P* ≤ 0.05) between experimental and control groups and double asterisks (**) mark the extremely significant difference (*P* ≤ 0.01). Error bars indicate SE (n = 3).

To research the effect of pcCpG on immunoglobulin *in vivo*, mRNA expression levels of CiIgM (Figure 8A), CiIgD (Figure 8B) and CiIgZ (Figure 8C) were measured by qRT-PCR at D0, D1, D5, D10, D14, D16 and D20 in spleen (Left) and head kidney (Right) tissues from grass carp immunized with vaccine or vaccine-pcCpG and challenged with GCRV at D15. The mRNA expression of CiIgM was extremely significantly induced by vaccine-pcCpG in spleen and head kidney comparing to other experimental groups and kept in high level at D10, D14 and D16. Meanwhile, the expression level in head kidney was higher than that in spleen. The change of CiIgD mRNA expression being suppressed by pcCpG remained to be further studied. There was no remarkable change of CiIgZ mRNA expression during the experimental period in spleen and head kidney of grass carp from all groups.

**Figure 9:**
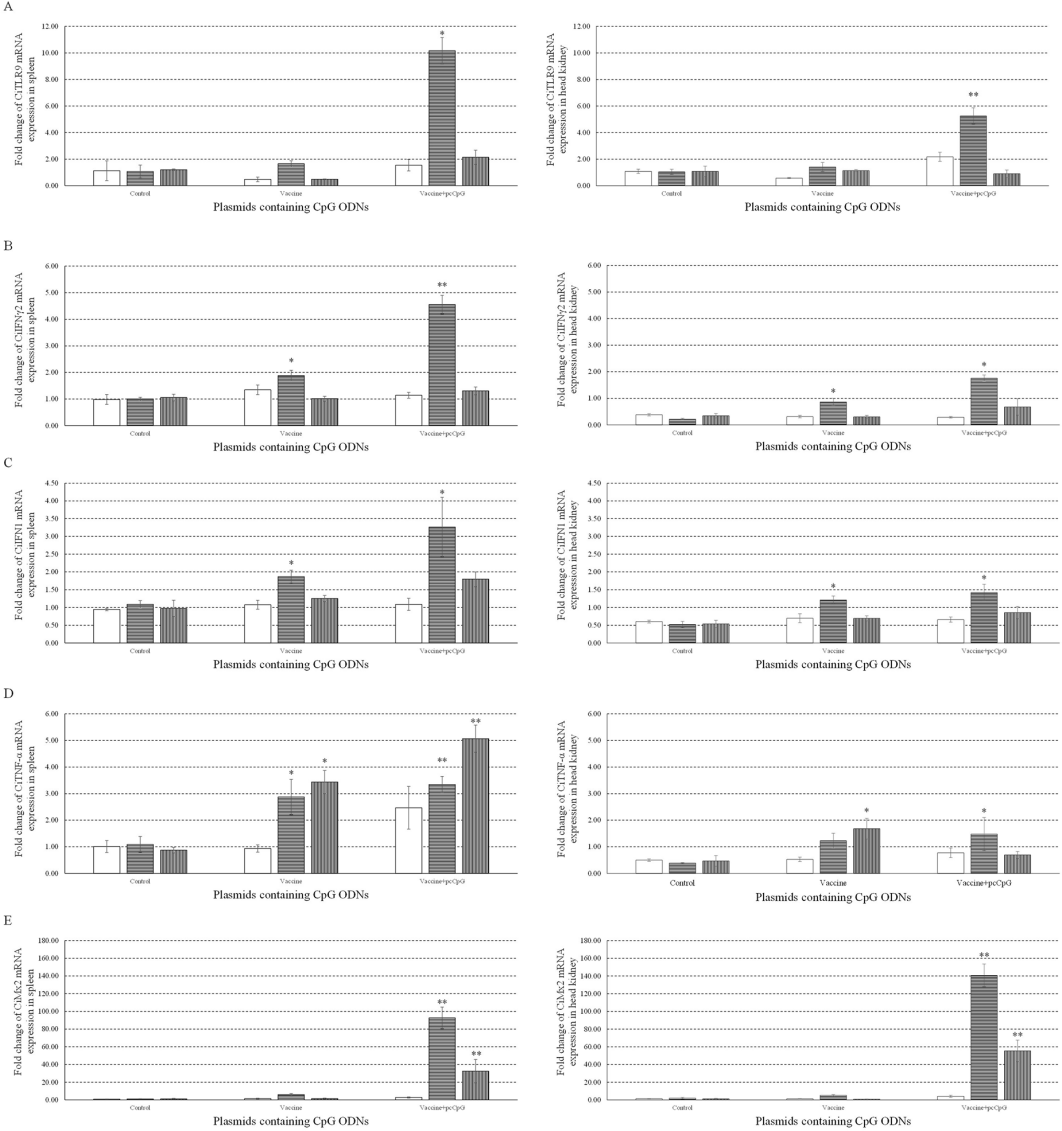
mRNA expressions of immune related genes individuals The mRNA expressions of CiTLR9 **(A)**, CiIFNγ2 **(B)**, CiIFN1 **(C)**, CiTNF-α **(D)** and CiMx2 **(E)** were measured at D0, D1, D10 and D16 post immunization in spleen (Left) or head kidney (Right). Grass carp were vaccinated with normal saline (NS), vaccine and vaccine-pcCpG respectively, and tissues were obtained from 5 random grass carp from each group at each time point. Other captions were the same as Figure 8.

Meanwhile, to investigate the immune responses to pcDNA3.1-1670A*5 *in vivo*, mRNA expression levels of CiTLR9 (Figure 9A), CiIFNγ2 (Figure 9B), CiIFN1 (Figure 9C), CiTNF-α (Figure 9D) and CiMx2 (Figure 9E) were measured by qRT-PCR at D0, D1, D10 and D16 in spleen (Left) and head kidney (Right) tissue from grass carp immunized with vaccine or vaccine-pcCpG and subsequently challenged with GCRV at D15. CiTLR9, CiIFNγ2, CiIFN1 and CiMx2 mRNA expression significantly increased in spleen and head kidney from grass carp immunized with vaccine-pcCpG obviously more than control or vaccine group and reached their peak levels at D10. CiTNF-α mRNA expression had a similar tendency but the peak level at D16. Meanwhile, the expression levels of CiTLR9, CiIFNγ2, CiIFN1 and CiTNF-α in spleen tissue were higher than in head kidney, but CiMx2 had the opposite condition. The result of CiIL-12 mRNA expression in spleen and head kidney was not shown because the inhibited expression could not be detected by qRT-PCR.

### pcCpG protects grass carp against GCRV infection

**Figure 10:**
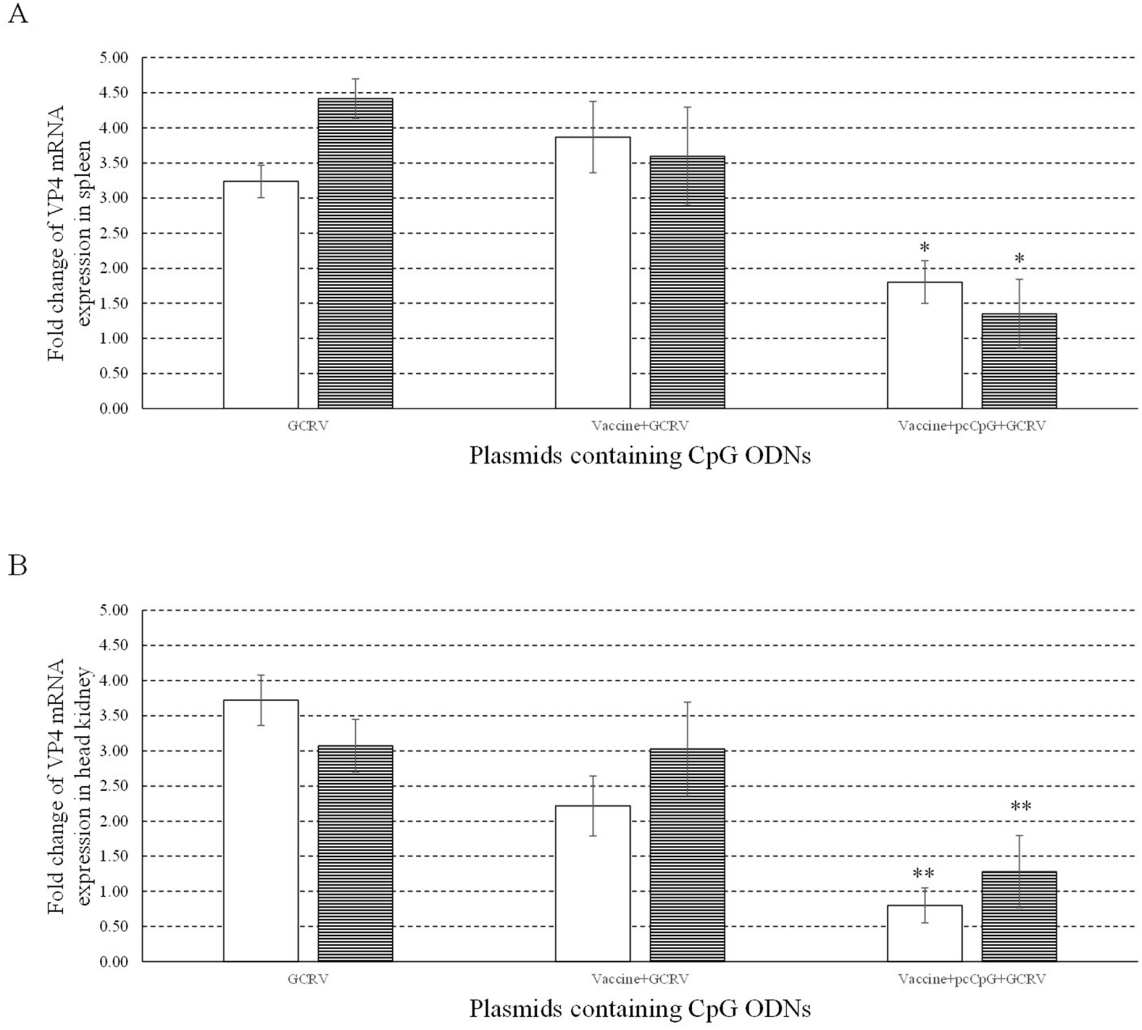
Antiviral activities of pcCpG *in vivo* The mRNA expression of VP4 was measured at 0, 1 and 5 d post infection in spleen **(A)** and head kidney **(B)**. Grass carp were vaccinated with normal saline (NS), vaccine and vaccine-pcCpG and infected with GCRV at D15 respectively, then spleen and head kidney tissues were obtained from 5 random grass carp from each group at each time point. Other captions were the same as Figure 8.

To investigate the immune protection and adjuvant function of pcCpG *in vivo*, VP4 mRNA expression was measured in spleen (Figure 10A) and head kidney (Figure 10B) from grass carp sampled at D0, D16 and D20. VP4 mRNA expression was respectively significantly and extremely significantly suppressed in spleen and head kidney of grass carp immunized with vaccine-pcCpG and subsequently infected with GCRV, demonstrating pcCpG could assist inactivated vaccine to be used as immune adjuvant to protect grass carp against GCRV infection.

## DISCUSSION

Our earlier study has demonstrated that CpG ODN 1670A was the optimal sequence for grass cap with high immune stimulatory activity by activating TLR9, RIG-I and IFNγ2-mediated signaling pathways [26]. CpG ODN 1826 and 1681 are efficient immunopotentiator reported in mice [29] and human [30], but were not as effective as 1670A in grass carp [26]. The present experiments address the question of the pragmatic function and the specific method of application of CpG adjuvant. The major object of this study was to find out a realistic efficient CpG adjuvant with intense antiviral activity usable in grass carp culture against GCRV infection.

To screen out the best plasmid containing CpG ODNs amongst the twelve recombinant plasmids and three empty vectors, CIK cells proliferation assay was performed and the result showed that pcDNA3.1-1670A*5 had the strongest proliferation enhancing activity. Meanwhile, three kinds of empty vectors also had proliferation enhancing activity to some degree, just as previous studies showing that plasmid DNA directly injecting into mice muscle cells could activate humoral and cellular immune responses [31]. Previous studies have demonstrated that the adjuvant property of plasmid DNA were dependent on its content of CpG motifs [32]. In this point of view, pcDNA3.1-1670A*5 contains 5 repeats of CpG ODN 1670A which including three CpG motifs. Thus, pcDNA3.1-1670A*5 with fifteen motifs had the best impact on CIK cells. After 24 h GCRV infection, CIK cells stimulated with pcDNA3.1-1670A*5 for 48 h remained the most number of cells while other groups all reduced by half or so indicating pcDNA3.1-1670A*5 could resist cell death caused by GCRV infection showing its antiviral activity. VP4 gene is segment 6 outer capsid protein of grass carp reovirus strain GCRV-097. VP4 expression represents GCRV replication. In CIK cells infected with GCRV after pcDNA3.1-1670A*5 vaccinated for 48 h, VP4 expression was significantly inhibited showing the antiviral activity just as the result of cell counting. Also in the crystal violet staining result with four repeats, the CIK cells vaccinated with pcDNA3.1-1670A*5 have stronger resistance to GCRV than other control (vaccinated with PBS) or experimental (vaccinated with empty vector or pcDNA3.1-1670A) groups while mock group without any treatment.

TLR9 is the specific receptor for CpG ODN [25]. pcDNA3.1-1670A*5 could activate TLR9 expression showing that it worked depending on the content of CpG motifs through TLR9-mediated pathway. Whereas, it did not activate RIG-I, a member in family RLRs (RIG-I-like receptors) which can sense viral PAMPs in both teleost and mammals [33, 34], which is different from the functionary mechanism of CpG ODN 1670A [26]. IL-2, a type of cytokine signaling molecule in the immune system, can act as growth factor of T cells subgroup, promotes B cells proliferation and also participates in antibody responses [35]. In consideration of the various functions of IL-2, pcDNA3.1-1670A*5 which did induce the IL-2 expression indeed had CIK cells proliferation enhancing activity and triggered antibody production in the follow-up results. NFκB1 and NFκB2 are critical members in NFκB family, controlling transcription of DNA, cytokine production and cell survival [36]. It plays a key role in antiviral immune responses. Incorrect regulation of NFκB has been linked to cancer, inflammatory and autoimmune diseases, septic shock, viral infection and improper immune development. NFκB has also been implicated in processes of synaptic plasticity and memory [37, 38]. pcDNA3.1-1670A*5 induced NFκB1 and NFκB2 mRNA expression in CIK cells implying that it activated NFκB pathway and downstream immune related genes and sequentially antiviral immune responses to GCRV infection.

IFN, the indispensable component of innate antiviral immunity, is the first line of host defense against virus infection. Mammalian IFN system consists of three types: type I IFNs, type II IFNs and type III IFNs [39]. Similarly to other fish, grass carp IFN system contains two types: type I IFN (IFN1, IFN2, IFN3 and IFN4) and type II IFN (IFNγ1 and IFNγ2) [40]. In the present study, CiIFN1, CiIFN3 and CiIFNγ2 gene expressions were detected after CpG vaccination and subsequent GCRV infection. CiIFNγ2 expression significantly increased by 17.67 folds at 48 h post-pcDNA3.1-1670A*5 vaccination and GCRV infection, while CiIFN1 and CiIFN3 just increased slightly by 2.13 and 5.47 folds comparing to control indicating the different functions between IFN-I and IFN-II, while CiIFN-II plays the main role during the process of antivirus against GCRV.

To confirm the adjuvant potential of pcDNA3.1-1670A*5 *in vivo*, immunization and GCRV infection were administered in grass carp individuals, and tissues were sampled for WB, ELISA, IHC and qRT-PCR analyses. The IgM expression of control group was found no significant difference, while in experimental groups, especially vaccine-pcCpG group, continuously raised and reached the peak at D10. Among different experimental groups, IgM expression level of vaccine-pcCpG group was the highest (Figure 5B) showing that the vaccine-pcCpG treatment was the most efficient immune therapy and pcCpG could be used as adjuvant assisting inactivated vaccine triggering adaptive immune. Also in the result of ELISA and IHC, vaccine-pcCpG was proved to have strong IgM expression inducing activity performing in the high ELISA index and high positive rate. To research the Ig expression systematically, qRT-PCR of IgM, IgD and IgZ was performed in spleen and head kidney tissues from grass carp at D0, D1, D5, D10, D14, D16 and D20, respectively. The IgM expression had almost the same tendency as the results of WB, ELISA and IHC, which was higher in head kidney corroborating the primary immune organ of head kidney. IgD expression was down-regulated and its functions remain to be further studied in teleost. IgZ expression level did not have any obvious change no matter in spleen or head kidney.

TLR9, IFNγ2 and IFN1 mRNA expression were also measured in spleen and head kidney tissues at D0, D1, D10 and D16. Vaccine-pcCpG immunization significantly induced the four genes expression and reached the peak at D10 indicating its immune response activating activity. TNF-α is a cell signaling protein involved in systemic inflammation and one of the cytokines that make up the acute phase reaction [41]. TNF-α expression significantly increased at D10 and D16 in spleen and at D10 in head kidney from grass carp immunized by vaccine-pcCpG. Inflammation was triggered in immunized grass carp and lasted for long term in spleen tissue showing the immune enhancing activity of pcCpG is stronger than vaccine treatment alone. Mx (myxovirus resistant) proteins are induced by IFN and can inhibit viral replication in various vertebrates [42]. Mx2, an effector molecule in antiviral process [43], was extremely significantly induced by over a hundred folds at D10 in spleen or head kidney from grass carp immunized with vaccine-pcCpG. VP4 expression was significantly reduced at the same time indicating the intense antiviral activity of vaccine-pcCpG treatment which was more efficient than vaccine immunization alone. These results showed the antiviral function of pcCpG and its potential to be used as adjuvant assisting inactivated vaccine protecting grass carp against GCRV in grass carp production and disease control.

In summary, plasmid containing CpG ODNs, pcDNA3.1-1670A*5, was screened out from twelve recombinant plasmids and three empty vectors by CIK cells proliferation activity assay. Moreover, dual luciferase reporter experiments of IFN and immune related genes expression by qRT-PCR in CIK cells showed that pcDNA3.1-1670A*5 had the strongest CIK cells proliferation activity and antiviral activity comparing to other fourteen plasmids and could specifically induce downstream signaling pathway gene expressions including TLR9, IL-2, NFκB1, NFκB2, IFNγ2, IFN1 and IFN3. These results demonstrated the potential of pcDNA3.1-1670A*5 as vaccine adjuvant. Further, the adjuvant function of pcCpG was investigated *in vivo* by IgM expression detection through western blotting, ELISA and IHC, and immune related genes expression (TLR9, IFNγ2, IFN1, TNF-α and Mx2) with qRT-PCR confirming that pcCpG could increase both innate and adaptive immunity. Meanwhile, the inhibited VP4 expression indicated its antiviral activity against GCRV. This study revealed that pcDNA3.1-1670A*5 can act as an efficient vaccine adjuvant assisting inactivated GCRV vaccine enlightening the application of CpG adjuvant in grass carp production and disease control. Moreover, these results provided abundant theoretical basis and experience for the research and employment of new type adjuvant CpG ODN and the second generation adjuvant in aquatic production disease control and livestock breeding industry.

## MATERIALS AND METHODS

### Experimental animals and cells

Grass carp were obtained from Honghu Wulin Seed Farm (Honghu, China) and kept in 1000 L tanks at 25°C with a constant flow of filtered water. Fish (15-25g) were fed with pellet food twice a day at 8:00 am and 5:00 pm with a daily ration of 0.7% of their body weight to be acclimated to feeding conditions for two weeks.

CIK (*C. idella* kidney) cell line is provided by China Center for Type Culture Collection and grown in DMEM (Gibco, USA) supplemented with 10% fetal bovine serum (FBS; Biosource, USA), 100 U/ml of penicillin (Sigma, USA) and 100 U/ml of streptomycin (Sigma, USA) according to the previous report [44]. CIK cells consist of many kinds of cells including fibre cells, polygon cells and giant cells in grass carp kidney [45] which is the major immune organ in fish. CIK cells were incubated at 28°C in a 5% CO_2_ humidified atmosphere.

### CpG ODN and plasmids construction

Synthetic ODNs are short single-stranded unmethylated DNA sequences. CpG ODN 1670A has been reported in our previous study. One, three or five repetitions of CpG ODN 1670A or combination with CpG ODNs 1826 and 1681 were synthesized and respectively inserted into pcDNA3.1 (+), pUC18 and pVAX1 [46] by Genwiz (Suzhou, China). The sequences of fragments and vectors were shown in Table 1. Recombinant plasmids were provided by puncture bacteria and powder and stored at -20°C.

**Table 1:** Fragments used for vector construction

Fragment name	Sequence (5’→3’)	Size (bp)
1670A	TCGAACGTTTTAACGTTTTAACGTT	25
1670A*3	TCGAACGTTTTAACGTTTTAACGTTTCGAACGTTTTAA CGTTTTAACGTTTCGAACGTTTTAACGTTTTAACGTT	75
1670A*5	TCGAACGTTTTAACGTTTTAACGTTTCGAACGTTTTAA CGTTTTAACGTTTCGAACGTTTTAACGTTTTAACGTTT CGAACGTTTTAACGTTTTAACGTTTCGAACGTTTTAAC GTTTTAACGTT	125
1826+1681+1670A	TCCATGACGTTCCTGACGTTACCGATGTCGTTGCCGGT GACGTCGAACGTTTTAACGTTTTAACGTT	67

### Cell proliferation assay

Briefly, 100 μl cell suspension was added into 96-well plates and incubated at 28°C humidified atmosphere, then stimulated respectively with PBS (control), empty vectors and recombinant plasmids containing CpG ODNs for 48 h, followed by infecting with GCRV for 24 h. the final CpG concentration was 5 μM. Then, cell concentration was detected by Countstar Automated Cell Counter (Countstar, China). Proliferation activity was proportional to the cell concentration.

### Stimulation and GCRV infection

CIK cells were cultured in 24-well plates, washed and counted by a hemocytometer and then re-suspended into a final concentration of 6 × 10^5^ cells/ml supplemented with 10% FBS. After 24 h, steadily cells were washed and cultured in DMEM without FBS. Then, they were stimulated with PBS (control), empty vector or plasmid containing CpG ODNs at a final CpG concentration of 5 μM. Cells were harvested at 1000 rpm for 8 min at 0, 24 and 48 h after stimulation. Samples of 0 h were collected without stimulation.

CIK cells were prepared as the method above. CIK cells stimulated with PBS (control) or plasmids for 48 h were infected with GCRV (097 strain, 3.63 × 10^7^ TCID_50_/ml) at a multiplicity of infection (MOI) of 1. Cells were harvested at 1000 rpm for 8 min at 0, 24 and 48 h after infection. Samples of control were collected without GCRV infection and those of GCRV control group were treated with GCRV infection only.

Total RNA of samples for qRT-PCR were extracted from cells with TRIzol Reagent (Aidlab, China) and its purity and quantity were measured using protein and nucleic acid analyser and agarose gel electrophoresis. mRNA was reverse-transcribed into cDNA with M-MLV reverse transcriptase (Promega, USA).

### Cytopathic effect assay

To confirm the antiviral activity of pcDNA3.1-1670A*5 in CIK cells, cytopathic effect (CPE) assay was carried out. CIK cells were seeded into 96-well plates with a concentration of 10^5^ cells/well overnight, then stimulated with PBS (control), pcDNA3.1 (+) empty vector, pcDNA3.1-1670A or pcDNA3.1-1670A*5 and subsequently infected with GCRV. At 24 h post-infection, cells were washed and fixed with 10% paraformaldehyde for 15 min at room temperature and stained with 0.05% (wt/vol) crystal violet (Sigma, USA) for 30 min, then washed with water and drained. Finally, the plates were photographed under a light box (Bio-Rad).

### Dual luciferase report system

CIK cells were seeded in 24-well plates and 24 h later co-transfected with IFN1/IFN3 indicated luciferase reporter plasmids and pRL-TK. pRL-TK vector (Promega, USA) was used as internal control to normalize the expression level of the transfected plasmid. At 16 h post-transfection, CIK cells were stimulated with PBS (control), empty vector plasmid or plasmid containing CpG ODNs for 16 h, followed by 12 h GCRV infection, and then washed with PBS and lysed by Passive Lysis Buffer (Promega, USA). Dual luciferase reporter assay was conducted in 96-well luminometer plates with Dual luciferase Reporter Assay System according to the manufacturer’s instructions (Promega, USA). Luciferase activity was measured by Multiscan Spectrum (PerkinElmer, USA). Data represent relative firefly luciferase activity normalized to Renilla luciferase activity. Results were obtained from four independent experiments and each was performed in triplicate.

### Vaccine and immunization

pcCpG was used to represent pcDNA3.1-1670A*5 in individual experiment. GCRV inactivated vaccine was kindly provided by Prof. Lingbing Zeng. Grass carp were randomly divided into 5 groups; control, vaccine, vaccine-pcCpG, pcCpG and GCRV. Tissue samples of control group were obtained without any treatment before immunization. Experimental grass carp were respectively immunized with normal saline (NS) (0.65% NaCl solution), vaccine, vaccine-pcCpG, pcCpG or normal saline at Day 0 (D0) by intraperitoneal injection (i.p.) and infected with 150 μL GCRV solution per fish except control group at Day 15 (D15) by i.p. Spleen and head kidney samples of each group were obtained at Day 1 (D1), Day 5 (D5), Day 10 (D10), Day 14 (D14), Day 16 (D16) and Day 20 (D20) except GCRV group. The samples of GCRV group were obtained 1, 5, 10 and 14 d after infection at Day 16 (D16), Day 20 (D20), Day 25 (D25) and Day 29 (D29) to contrast with samples of other groups at D1, D5, D10 and D16. The specific dosage of recombinant plasmid was 15 μg per fish and inactivated vaccine was 200 μl per fish according to previous researches [47, 48]. Methods of tissue total RNA extraction and reverse transcription were the same as those of cells.

### IgM expression analysis

To detect the expression of IgM in grass carp serum, western blotting (WB), enzyme linked immunosorbent assay (ELISA) and immunohistochemical (IHC) were performed. For WB, sample serum was diluted 10 times and 10μL of serum protein extracts separated on 10% sodium dodecyl sulfate-polyacrylamide gel electrophoresis (SDS-PAGE) gels and transferred onto a NC membrane (Millipore). The membrane was blocked in 2% bovine serum albumin (BSA) dissolved in TBST buffer for 2 h at room temperature, then incubated with mouse monoclonal antiserum of CiIgM for 2 h at room temperature. The antiserum was kindly provide by Prof. Pin Nie. It was subsequently incubated with secondary antibody for 45 min at room temperature. IRDye^@^ 800CW Donkey anti-mouse-IgG (H+L) secondary antibody was purchased from LI-COR. Signals were visualized with Odyssey Clx (Li-COR Biosciences, USA).

For ELISA, 96-well plates were coated with grass carp serum sample in 100 μl NaHCO_3_ in each well at 4°C overnight. After all of the coated wells were blocked with 2% BSA, mouse monoclonal antibody of CiIgM was loaded into the wells and incubated at 37°C for 1 h. Anti-E-tag mouse monoclonal antibody with HRP conjugate (1:10000 dilution in TBST, Amersham Biosciences, Sweden) was then added and incubated at 37°C for 1 h. Finally, 3,3’, 5,5’-tetramethylbenzidine (Serva, Germany) was used for color reaction, which was stopped with H_2_SO_4_. Absorbance was determined at 450 nm using a spectrophotometer (BioTek, USA).

For IHC, three random grass carp spleen tissues were sampled and fixed with 4% paraformaldehyde over 24 h used for sample presentation. IHC was performed by Biofavor (Wuhan, China).

### qRT-PCR

Roche LightCycler^®^ 480 system was used to quantify the mRNA expression of the listed genes followed by their GenBank accession number: CiTLR9 (FJ969850), CiRIG-I (GQ478334), CiIFN1 (DQ357216), CiIFN3 (KU182642), CiIFNγ2 (AGQ16237), CiIL-2 (AF486820), CiIL-12 (KF944668), NFκB1 (nuclear factor-kappaB 1) (KY613788), NFκB2 (KY613789), VP4 (GQ469997), CiIgM (DQ417927), CiIgD (GQ429174), CiIgZ (GQ201421), CiTNF-α (HQ696609), CiMx2 (JF699168) and endogenous reference (EF1α, GQ266394 and 18S rRNA, EU047719) using BioEasy Master Mix (SYBR Green) (Hangzhou Bioer Technology Co., Ltd, China). Melting curve analysis of amplification products was performed to confirm that only one PCR product was amplified and detected. The primers were listed in Table 2. The comparative cycle threshold (CT) method (∆∆CT) was used to quantitate the expression of target genes that was normalized to EF1α/18S rRNA expression levels of transcripts from uninfected control cells/tissues [49]. All data are given in terms of mRNA levels relative to those of the internal reference and expressed as means plus or minus standard errors of the mean (SE).

**Table 2:** Primers used for qRT-PCR analysis

Primer name	Sequence (5’→3’)	Gene name	Size (bp)
EF125	CGCCAGTGTTGCCTTCGT	EF1α	99
EF126	CGCTCAATCTTCCATCCCTT		
TF155	CAGTTGCGTTATCTCGGGGT	TLR9	217
TR156	CTTCAGGAGGGGAATGATGGT		
RF230	ACTACACTGAACACCTGCGGAA	RIG-I	70
RR231	GCATCTTTAGTGCGGGCG		
VF146	CGAAAACCTACCAGTGGATAATG	VP4	135
VR147	CCAGCTAATACGCCAACGAC		
ILF1702B	CTTTGTCGGGGTCCTAATTATGT	IL-12	159
ILR1552	GTGCTTTTGCTTTGATGATGGA		
IF750	AGCCTGTGAAACGAAGCATC	IL-2	136
IR751	GAAGACAAACAATCCTCCTGAAT		
IF590	AAGCAACGAGTCTTTGAGCCT	IFN1	79
IR591a	GCGTCCTGGAAATGACACCT		
IF435	TACATTTATAGAGACTGCGGGTGG	IFN3	144
IR357	TGGAGTGTCTGGTAAACAGCCTT		
WF79	CAGCGAACACCTGAAACTAACA	IFNg2	88
WR80	CCATCCCAAAGTCATCAAACAT		
NF610	CCAGGTGCGGTTTTATGAAGATGA	NFκB1	200
NR611	ATGGCTTGGGTTCGCTCGTTT		
NF612	AGGCACTTCCTCAGCACTACGAT	NFκB2	110
NR613	AAAACCTCCTCCCATTCCACC		
18F99	ATTTCCGACACGGAGAGG	18SrRNA	90
18R100	CATGGGTTTAGGATACGCTC		
IgF899	GTCTACCTCCAACTCCACCACC	IgM	187
IgR900	GTGTTTATTGTATTTGCCACCTGA		
IgF901	TGGCAACTAAATGGGACGAA	IgD	174
IgR902	GTTAAATGGACTTTGGGATGACTC		
IgF909	ATAAAGGAGATGAAAAGACACCCA	IgZ	204
IgR910	TGCTGACAACCGATGTGGAG		
TnfF169	GCTGCTGTCTGCTTCACGC	TNF-α	189
TnfR170	AGCCTGGTCCTGGTTCACTCT		
MF428	ACATTGACATCGCCACCACT	*Mx2*	129
MR429	TTCTGACCACCGTCTCCTCC		

### Statistical analysis

The data were analyzed using an unpaired, two-tailed Student's t-test. *P* values below 0.05 were regarded as being significant for all analyses (*, *P* ≤ 0.05; **, *P* ≤ 0.01). Experiments were repeated four times.
